# Barriers and enablers to implementation of the therapeutic engagement questionnaire in acute mental health inpatient wards in England: A qualitative study

**DOI:** 10.1111/inm.13047

**Published:** 2022-08-17

**Authors:** Francesca Taylor, Sarah Galloway, Kris Irons, Lorna Mess, Laura Pemberton, Karen Worton, Mary Chambers

**Affiliations:** ^1^ Joint Faculty of Health, Social Care and Education Kingston University and St George's University of London London UK; ^2^ South‐West London and St George's Mental Health NHS Trust London UK; ^3^ Priory Group London UK; ^4^ North‐East London NHS Foundation Trust London UK; ^5^ Southern Health NHS Foundation Trust London UK; ^6^ Cumbria, Northumberland, Tyne and Wear NHS Foundation Trust London UK

**Keywords:** COVID‐19, inpatient care, mental health nursing, therapeutic engagement, therapeutic intervention

## Abstract

A strong association exists between the quality of nurse‐service user therapeutic relationship and care outcomes on acute mental health inpatient wards. Despite evidence that service users desire improved therapeutic engagement, and registered mental health nurses recognize the benefits of therapeutic relationships, such interactions remain sub‐optimal. There is a dearth of evidence on factors influencing implementation of interventions to support and encourage therapeutic engagement. This study aimed to understand the barriers and enablers to implementation of the Therapeutic Engagement Questionnaire (TEQ), across fifteen acute inpatient wards in seven English mental health organizations. Qualitative methods were used in which data were collected from ethnographic field notes and documentary review, coded, and analysed using thematic analysis. Theoretical framing supported data analysis and interpretation. Reporting adheres to the Standards for Reporting Qualitative Research. The TEQ as an evidence‐based intervention co‐produced with service users and nurses was valued and welcomed by many nurse directors, senior clinicians, and ward managers. However, a range of practical and perceptual factors impeded implementation. Furthermore, many existing contextual challenges for intervention implementation in acute inpatient wards were magnified by the COVID‐19 pandemic. Suitable facilitation to address these barriers can help support implementation of the TEQ, with some transferability to implementation of other interventions in these settings. Our study suggests several facilitation methods, brought together in a conceptual model, including encouragement of reflective, facilitative discussion meetings among stakeholders and researchers, effort put into winning nurse ‘buy‐in’ and identifying and supporting ward‐level agents of change.

## INTRODUCTION

Improving service user (SU) experience of acute mental health inpatient care is a prominent concern for many clinicians, mental health SU‐led organizations, policymakers, and researchers worldwide (Csipke *et al*. 2016; Delaney *et al*. 2020). Inpatient care remains an essential element of most countries' mental health systems although they work predominantly towards a community‐based care model (OECD 2021; Patel *et al*. 2016). Reflective of admission criteria, individuals admitted as inpatients are generally experiencing the most acute mental health issues, requiring high quality care in a safe environment (Delaney *et al*. 2020). Yet an inhospitable picture of the acute inpatient ward environment is manifest in the literature. SUs report their ward experience as lacking warmth, respect, treatment choice, and person‐centred care (Cutliffe *et al*. 2015; Staniszewska *et al*. 2019), while being managed ‘as problems to be solved’ (Moreno‐Poyato *et al*. 2016). A significant and consistent criticism is the absence of therapeutic engagement and interpersonal therapeutic relationships (Cutliffe *et al*. 2015; McAllister *et al*. 2021a).

Over the past two decades, UK and international policy has highlighted the importance of integrating nurse–patient therapeutic communication and engagement into acute mental health inpatient care (Department of Health and Social Care 2021; World Health Organization 2013). The quality of therapeutic relationship between nurse and SU in this setting is strongly associated with care outcomes (Hartley *et al*. 2020). SUs value therapeutic engagement, perceiving it to be an essential component of recovery‐focused care and contributing to feelings of trust and safety (Cutler *et al*. 2020; Staniszewska *et al*. 2019).

Furthermore, therapeutic engagement has been considered the crux of mental health nursing (Chambers 1998) since the publication in 1952 of Peplau's seminal work which emphasized the primacy of the nurse–patient relationship (Peplau 1952). Nurses on acute inpatient wards are well placed to develop therapeutic relationships as SUs interact with them for the largest proportion of time (McAndrew *et al*. 2014). Many nurses want to engage therapeutically with SUs and seek opportunities to do so, recognizing the considerable benefits (Cleary *et al*. 2012; Delaney *et al*. 2017). However, the ability to do this effectively is mediated often by professional challenges such as confidence, knowledge, and capacity, alongside workforce issues such as time constraints, staff‐patient ratios, and crisis handling (McAllister *et al*. 2021a). Studies suggest that nurses working in time‐pressured, risk‐focused acute inpatient environments tend to prioritize administration issues and tasks over therapeutic support (Wykes *et al*. 2018; Kingston & Greenwood 2020).

Additional challenges include lack of clarity around the different variables that can contribute to therapeutic engagement (Chambers *et al*. 2017) and an absence of shared descriptive language, compounded by nurses often struggling to articulate what they do in therapeutic interchange with SUs (McAllister *et al*. 2021a). Given its tacit nature, the therapeutic practice of nurses can go unrecognized (Hartley *et al*. 2020; Hurley *et al*. 2020). Another constraint is the dearth of evidence‐based interventions reported to support nursing staff in therapeutic engagement, with few successfully implemented into practice (McAllister *et al*. 2021a, b). Interventions that have been implemented consist mainly of group‐based programmes, whereas the predominant therapeutic engagement relationship is dyadic between one nurse and one SU. The involvement of SUs and/or nurses in the development of the interventions is poor (Hartley *et al*. 2020). Such involvement might have increased the interventions' acceptability and impact (Brett *et al*. 2014).

## BACKGROUND

Given the clinical importance of therapeutic engagement and its perceived benefits from the perspective of registered mental health nurses (RMHNs) [registered as fit to practice as mental health nurses] and SUs, there is value in measuring the nature of nurse‐SU therapeutic interactions from both viewpoints (Chambers *et al*. 2017). Prior to development of the TEQ, there was no such quantitative tool available. Unwarranted variation in mental health nursing practice could arise, as it was not possible to apply objective measures consistently (McAndrew *et al*. 2014). There was also no means to capture the conceptual complexity of therapeutic engagement (Moreno‐Poyato *et al*. 2018; McAllister *et al*. 2019) and therefore give recognition to the contribution made by RMHNs to SU recovery (Chambers *et al*. 2017).

The TEQ was the first intervention in acute inpatient settings to be co‐produced with SUs and nurses. Developed in three stages – item generation, item reduction, and validation – it is psychometrically sound and valid as a measure of therapeutic engagement. Full details of the development of the TEQ are provided in earlier published articles (Chambers *et al*. 2017, 2019).

Evidence‐based health care is accepted widely as a quality standard of mental health practice (Le Boutillier *et al*. 2015). Nonetheless, the acute mental health inpatient setting presents particular and significant challenges to successful implementation of evidence‐based interventions (Raphael *et al*. 2021; Sandstrom *et al*. 2015). To create an effect, an intervention needs to be adopted, impact on beneficiaries, and result in behaviour change. Yet the distinct social processes and contextual features that characterize acute inpatient wards provide constraining barriers to change; limited resources particularly staff and beds, fast discharge, and risk‐averse cultures with high potential for disruption through violence and aggression (Laker *et al*. 2014; Raphael *et al*. 2021). In this demanding context, involvement of ward staff in the development and assessment of an intervention implementation process is apposite, to help provide suitable fit of the intervention to a specific ward environment (Laker *et al*. 2019; Hartley *et al*. 2020).

An understanding of how evidence‐based interventions are implemented into practice is critical to effectiveness in health systems (Boaz *et al*. 2016). An effective implementation strategy requires appreciation of the enablers as well as the barriers associated with the implementation process (Forsner *et al*. 2010). However, there is limited evidence on the elements that can influence successful implementation of therapeutic engagement interventions in acute mental health inpatient ward settings (McAllister, 2021a). Our study seeks to address this evidence gap by examining the factors that enable or hinder implementation of the TEQ.

To understand better the process of implementation and the interplay of facilitators and barriers, we used the Promoting Action on Research Implementation in Health Services (PARIHS) as a framework for the study. Developed by Kitson et al. (1998), and refined over time, the PARIHS was devised to help understand the complexities inherent in successful implementation. It conceptualizes success as a function of the qualities of the nature and type of research behind the innovation [evidence], the context where implementation is taking place [context], and the way implementation is facilitated [facilitation]. These three concepts were distinguished as the core elements needing attention in the implementation process. The PARIHS has been used in a variety of healthcare settings including mental health (Bergström *et al*. 2020). The aim of this study was to understand the barriers and enablers to implementation of a therapeutic engagement measurement tool, the TEQ, in acute mental health inpatient settings. Specifically, how can barriers be overcome to enable successful implementation of the TEQ?

## METHODS

### Design

The study design was in the interpretive tradition underpinned by a pragmatist approach, to enable focus on understanding of real‐world issues and production of actionable knowledge of practical relevance (Kelly & Cordeiro 2020). Qualitative methods were employed, including data from ethnographic field notes and documentary review (Holloway & Galvin 2016), to understand better the full nature of the barriers and enablers associated with implementation. Guidance for ensuring quality when undertaking qualitative research (O'Brien *et al*. 2014) was used to assess transparency in designing an appropriate methodological approach, trustworthiness of data collection and analysis, and extent of reflexivity. It was a multi‐site study across different regions of England, involving six NHS Mental Health Trusts and one private provider of mental health care facilities. Study sites volunteered to implement the TEQ in response to a letter sent by the study Principal Investigator through the National Mental Health Nurse Directors Forum. A total of 15 acute inpatient mental health wards implemented the intervention.

The form that implementation takes is better varied as required by context (Braithwaite *et al*. 2018) and therefore was not standardized. Two study researchers, FT and MC, provided support to the stakeholders in each study site during the implementation process. The support mechanism was facilitative discussion meetings involving the researchers and key stakeholders. Implementing organizations made the decisions on how often and when they would like the meetings and which stakeholders to invite. Stakeholders included nurse directors, senior clinicians (nurse consultants, quality improvement and innovation managers, matrons, Band 8 nurses [a high‐level National Health Service grade and salary scale]), ward managers, and nurses. SUs were not included in the facilitative discussion meetings. Implementing organizations used community meetings to discuss the TEQ implementation process with SUs and to hear SU views and recommendations about how and when the TEQ should be completed.

A total of 34 facilitative discussion meetings were held during the study, all online. The number of meetings with each implementing organization varied from three to ten. Each meeting lasted between one and one and a half hours.

The TEQ was implemented as an evaluated service intervention. As this study was part of local service evaluations to understand staff attitudes and opinions, ethical approval was not required. The TEQ intervention was signed off by the Nursing Directors of the implementing organizations, and the study was carried out in accordance with the principles of the Declaration of Helsinki.

Considerable attention was given by each organization implementing the TEQ intervention to ensure safety for the SUs and RMHN. This included providing assurances of anonymity, and ward managers followed by senior nursing staff checking the completed anonymous questionnaires for any safeguarding issues, and responding to any concerns raised.

### Intervention

The ‘hard core’ components of the intervention (Greenhalgh *et al*. 2004) are the RMHN and SU versions of the TEQ. In each version, therapeutic engagement is scored across two different contexts: one‐to‐one RMHN and SU interactions, and overall environment and atmosphere on the ward. Twenty therapeutic engagement statements are included with pre‐set answer options using a 4‐point Likert response scale. All questionnaire items require completion. Completion is done with anonymity. There is a specific scoring system attached to the TEQ. The higher the score the better the therapeutic engagement. Table 1 shows an exemplar SU and RMHN statement for the same TEQ item in relation to the environment and atmosphere on the ward, to illustrate how the statements vary. The rating scale is also shown.

**TABLE 1:** Exemplar service user and registered mental health nurse therapeutic engagement questionnaire statement

No.	Service user statement	Environment and atmosphere on the ward as created by the nursing staff			
		1	2	3	4
		Strongly disagree	Disagree	Agree	Strongly agree
	The nursing staff…				
1	Show me respect at all times				

No.	Registered mental health nurse statement	Environment and atmosphere on the ward			
		1	2	3	4
		Strongly disagree	Disagree	Agree	Strongly agree
	On the ward…				
1	We show SUs respect at all times				

The SU version of the TEQ should be completed by SUs at the time of their discharge from an acute mental health inpatient ward. They should be aged 18 years or over, have good command of the English language and the mental capacity to consent to complete the questionnaire, and had at least six care‐plan (recovery focused) interactions with their named/primary nurse. If help is needed to complete the questionnaire, they can ask a family member or relative, but not ward staff. The nurse version should be completed by RMHNs with a permanent work contract, working on adult acute inpatient wards. They should complete the TEQ at the time of discharge of a SU with whom, as their named/primary nurse, they have had at least six care‐plan (recovery focused) interactions.

### Data collection

The primary data collection method was ethnographic field notes collected during the facilitative discussion meetings. Two researchers attended and helped facilitate the meetings with stakeholders in each study site. One of these researchers simultaneously participated in the meetings while jotting down notes on dialogue, atmosphere, behaviour, and decisions. These jottings were expanded later into field notes that also included the researcher's reflections and feelings. Field notes were recorded from 41 h of meetings. In addition, requests were made to stakeholders of organizations implementing the TEQ for available documents on the process. These included facilitative discussion meeting notes, and presentation slides from internal staff presentations and national conferences. The number of documents made available by each implementing organization varied from one to three. The documents (*n = 15*) were reviewed for any evidence of commentary, observations or reports of factors influencing implementation of the TEQ. Data were collected between June 2020 and October 2021. All data were anonymized, and safe storage ensured in line with best practice principles of data protection and archiving.

### Data analysis

Data from the ethnographic field notes and documentation were imported into Excel and analysed collectively using thematic analysis (Pope *et al*. 2020). The PARIHS was used as an organizing framework for analysis and interpretation. An ongoing, iterative and constant comparative approach was employed to code and organize data into categories. Where data did not fit generated themes, new codes were developed or existing ones refined, until all data were coded. Memos were written throughout data analysis, providing detail of thoughts, considerations, and revisions as new data were added. This reflexive process (Boyatzis 1998) was undertaken independently by one researcher supplemented by collaborative discussion with the second researcher to reach consensus. These discussions were also used to consider reflexivity and how to minimize the influence of the researchers' experiences, beliefs, and values. A further stage of synthesis was undertaken after sharing emergent themes, to describe and interpret findings, looking for patterns and plausible explanations across data before confirmation of themes.

## RESULTS

Below we discuss the identified themes in relation to the core elements for successful implementation featured within the PARIHS framework: evidence, context, and facilitation. Table 2 shows the implementation elements and study themes. Table 3 highlights the barriers and enablers to implementing the TEQ within each core element of the framework.

**Table 2:** Core implementation elements and study themes

Implementation elements	Study themes
1. Evidence	Philosophy of the TEQ Fit with organizational goals Therapeutic engagement know‐how
2. Context	COVID‐19 pandemic challenges Data collection
3. Facilitation	Reflective and facilitative conversations Therapeutic engagement training and learning Ward‐level agents of change

**TABLE 3:** Summary of barriers and enablers to implementing the Therapeutic Engagement Questionnaire

PARIHS element (Kitson *et al*. 1998)	Barriers	Enablers
Evidence	Low motivation to advance recognition of RMHN role in SU recovery Low motivation to improve RMHN‐SU therapeutic engagement Inadequate nursing skills and knowledge to deliver against TEQ statements	Robust evidence base for TEQ Trust in how and by whom TEQ developed TEQ statements based on experiential perspective of SUs and nurses Statements show explicitly what therapeutic engagement involves TEQ data can give recognition to role of RMHNs in SU recovery TEQ complements current macro‐level policy initiatives TEQ data provides underpinning evidence for organizations' strategic and quality improvement initiatives
Context	Service impacts of COVID‐19 High rates admission and discharge Increased acuity and complexity of admissions SUs and staff needing to self‐isolate Depleted staff numbers/increased use of agency staff Task oriented culture TEQ perceived an administrative burden and stress contributor TEQ perceived as performance monitor TEQ in paper not app‐based format	Flexibility of TEQ, enabling adaptation to local site need: ○ when completed ○ number of therapeutic interactions necessary before completion Ward managers addressing nurse concerns Ward mangers explaining anonymity of TEQ data to nurses Ward managers discussing anonymised TEQ outcomes in staff meetings and how they demonstrate value of the RMHN role
Facilitation	Senior stakeholders/researchers discouraging discussion of barriers Senior stakeholders/researchers responding judgementally to barriers raised Senior stakeholders/researchers shutting down discussion of new ideas for implementation practice Absence of local TEQ implementation champion/s Departure of ward‐level agents of change without successor	Site‐based facilitative discussion meetings with stakeholders and researchers: ○ informal and non‐hierarchical ○ reflective and ‘think‐aloud’ ○ supportive and empathetic ○ story sharing of successful implementation practice Senior stakeholders open to change and overcoming local barriers Ward‐level agents of change TEQ used as a therapeutic engagement training resource SUs introduced to TEQ at admission ‘You said, we did’ ward noticeboard publicity for actions in response to TEQ findings

### Evidence

#### Philosophy of the TEQ

Across the study sites many stakeholders seemed motivated by the underlying philosophy of the TEQ, to give recognition to the role and contribution of RMHNs in SU recovery. The historical association of mental health nursing with therapeutic engagement was commented on by several nurse directors/senior clinicians. Acceptance of the clinical importance of therapeutic engagement was also manifest. Stakeholders reported RMHNs, particularly those working in the role for longer, felt that recognition of their value had diminished over time. This ‘invisibility’ was equated by some, with an absence of metrics. One stakeholder commented that ‘For audits it is a struggle to find evidence of what registered mental health nurses are doing’. (ID 02). Some nurse directors/senior clinicians described seeing potential for the TEQ to catalyse an improved awareness and understanding of the value of the profession in tandem with more prominence given to therapeutic engagement.

I was keen to do this [*implementation*] as demonstration of what nurses do. That was the appeal to me. This is about what nurses do. (Facilitative discussion meeting – Nurse Director/Senior Clinician, ID 01)

While the intervention appeared compatible with the values of most of those tasked with implementation on the wards, there were a few stakeholders for whom the intervention did not seem to accord with their values. They indicated less motivation to enhance therapeutic engagement and advance recognition for their profession and less engagement with implementation.

#### Fit with organizational goals

Senior stakeholders (Nurse Directors and Senior Clinicians) from many of the study sites expressed a belief that implementation of the TEQ would both complement and help underpin their individual organization's strategic priorities. They described feeling confident about using the intervention because of its robust evidence base and trust in how, and by whom, the TEQ was developed.

Some nurse directors/senior clinicians talked in terms of the TEQ supplementing quality improvement initiatives. One commented, for example, that ‘The TEQ can feed into the trust's quality improvement plan. We've just started an appreciative inquiry and the TEQ would fit very well into that’. [Facilitative discussion meeting – Nurse Director/Senior Clinician, ID 06]. Another mentioned using the TEQ as a pre and post measure to assess the impacts of practice changes to improve SU ward experience. Another referenced the role of the TEQ in supporting organizational strategic plans for change:

The TEQ supported our proposed new nursing staffing model to increase numbers of band 6 [band is the English National Health Service word for a pay and salary scale] experienced staff and a clinical band 7 on each ward, to support quality, enhance the nursing profession and support therapeutic engagement. (Document – Nurse Director/Senior Clinician, ID 04)

The strategic benefits of the TEQ were also recognized at individual ward level. For example, in one site, it was mentioned that the intervention would support accreditation efforts:

Some wards are aiming for accreditation and will use the TEQ to see how feedback from SUs is used. (Facilitative discussion meeting – Nurse Director/Senior Clinician, ID 07)

Some nurse directors/senior clinicians referenced the national picture and talked about the TEQ being complementary to current macro‐level policy initiatives on acute mental health inpatient care. For example, the Care Quality Commission (CQC) initiative to ensure safe and therapeutic inpatient ward care environments. There was some intent to make TEQ metrics a component of their reports or updates to national organizations. Additionally, a few senior stakeholders discussed the potential to link TEQ metrics with priority organizational quality and safety outcomes to provide more robust evidence for decision‐making.

#### Therapeutic engagement know‐how

Many stakeholders commented on the value of the statements incorporated in the TEQ because they explicitly show what is involved in therapeutic engagement. The statements were reported to give coherent meaning and understanding to what was needed by nurses to engage therapeutically with SUs. The statements were also said to provide a meaningful shared language based on the experiential perspective of SUs and nurses.

However, from the outset some stakeholders expressed concerns that not all nurses would have the skills to deliver the stated therapeutic engagement activities in practice. These concerns proved apposite. The engagement of some nurses in implementing the TEQ was reported to be restricted by their perceived lack of therapeutic engagement skills.

### Context

#### COVID‐19 pandemic challenges

The most frequently discussed challenge in all sites was the COVID‐19 pandemic. Intervention implementation and prioritization were reported to be hindered by a range of pandemic‐associated ward environmental factors, including high rates of admission and discharge, increased acuity and complexity of admissions, requirements to self‐isolate, depleted permanent staff numbers, and use of agency staff. The main service adaptation mentioned was the creation of specific wards for inpatients with confirmed or suspected infection. In some sites, stakeholders talked about reduced face‐to‐face contact because of infected inpatients being confined to their room.

The ward is acting as a direct admission ward where SUs are tested for COVID before being transferred. This requires SUs to isolate in their room until test results are received which can impact on therapeutic engagement. (Facilitative discussion meeting – Ward manager, ID 13)

Concerns were frequently raised about faster SU turnover and lack of protected engagement time constraining named/primary nurses' capacity to deliver six care‐plan interactions before SU discharge. Therefore, researchers and stakeholders agreed to modify this intervention component, SUs needing to have only a minimum of three care‐plan interactions with their named/primary nurse to complete the TEQ.

Another intervention component reported difficult to implement was the requirement for SUs and their named/primary nurse to complete the TEQ on the day of discharge. Stakeholders talked about nurses having insufficient time or capacity to do this and that some SUs had refused to complete the TEQ because of delayed discharge.

SUs refusing to complete the TEQ is due to frustration at delays in their discharge due to problems with accommodation. (Facilitative discussion meeting – Nurse Director/Senior Clinician, ID 01)

Hence the researchers and stakeholders agreed to more flexibility; nurses and SUs could complete the TEQ as close as possible to discharge day.

#### Data collection

Overall, data collection appeared sporadic. All sites reported receiving returned data but not consistently, i.e., at discharge of each SU. In several sites, stakeholders identified the need for routinely collected data as a challenge to sustained implementation. Ward managers mentioned that some nurses perceived regular completion of the TEQ as an additional administrative burden and potential contributor to stress and exhaustion.

Some nurses were reported to be reluctant to engage with the intervention because they perceived it as data collection to monitor individual performance, with potential negative consequences linked to criticism of existing practice.

Difficulties in getting the registered nurses to complete whereas the SUs are willing to complete…there is some suspicion as to whether it is a monitoring measure. (Facilitative discussion meeting – Ward manger, ID 09)

Methods employed to address these concerns and highlighted as particularly effective by ward managers, included, stressing the anonymity of data collected, discussing anonymised data outcomes in staff meetings, emphasizing the value of the data in demonstrating the RMHN contribution to SUs' recovery, and explaining the TEQ's improvement role.

It has been sold not as a tick‐box exercise but as being interested in what staff have to say…about improving the culture not a top‐down approach…it is made clear that the TEQ is about improvement not a monitoring process. (Facilitative discussion meeting – Ward manager, ID 15)

The paper format of the TEQ proved another challenge. Some stakeholders expressed the view that an app‐based version of the TEQ would facilitate implementation by making data collection more flexible and less time‐consuming.

### Facilitation

#### Reflective and facilitative conversations

Facilitative discussion meetings were initially set up in each site to discuss practical implementation approaches. Over time in many sites, these meetings evolved iteratively to become more reflective and conversational in nature, and the fulcrum by which implementation was adapted to local circumstances. In partnership, researchers and stakeholders reflected on what had been happening, what was working well and not working well, including organizational‐specific factors impeding implementation and how these might best be addressed. Story sharing of successful ward‐level implementation practice within sites featured highly. The researchers also shared (anonymously) practice that had worked well in other sites.

These conversations appeared especially valued by some stakeholders given the messy and complex inpatient environment amidst the COVID‐19 pandemic. Where a particularly supportive and empathetic conversational space was created, it seemed to give permission to discuss more emotional as well as practical implementation barriers. For example, how the negative impacts of the pandemic on nursing practice, including therapeutic engagement, were generating emotional anxiety, distress and burden:

Nurses feel guilty about not doing the things they should do. (Facilitative discussion meeting – Nurse Director/Senior Clinician, ID 04)

Conversations seemed most facilitative of implementation when nurse directors/senior clinicians openly encouraged ward staff to speak about the barriers as well as the enablers experienced. Where there was a sense of softened power structures, ward staff appeared more able and confident to raise issues. A non‐judgemental response from the researchers also seemed to encourage stakeholders towards open‐mindedness and creativity in suggesting facilitative changes. Conversations appeared to work less constructively if nurse directors/senior clinicians or researchers shut down comment and discussion.

#### Therapeutic engagement training and learning

Individual ward managers across different sites described using the TEQ to provide staff therapeutic engagement training. Utilization of the TEQ to improve therapeutic engagement skills, not just for measurement, was reported to foster stronger nurse engagement with the intervention. For example, one ward manager talked about using the TEQ's statements to demonstrate to nurses the specific aspects of therapeutic engagement they should be delivering:

I'm using the TEQ in clinical supervision sessions – “when working with SUs you can make sure you deliver these types of therapeutic support. This is the difference about being a registered mental health nurse.” (Facilitative discussion meeting – Ward manager, ID 12)

Another ward manager described monthly skills training sessions with nurses in which anonymized TEQ data were shown and discussed. This was said to encourage a sharing of ideas about how improvements might be made in relation to statements receiving lower scores from SUs.

#### Ward‐level agents of change

In some sites, senior stakeholders assigned individuals to champion implementation, while champions also emerged organically. Interestingly, it was the latter who gave the impression of being the more active and enthusiastic agents of change. Mostly ward managers, they appeared to share a strong belief in the value of therapeutic engagement's contribution to service improvement. They also seemed to have the creative and motivational drive to overcome implementation barriers.

Successful ideas that emanated from these ward‐level change agents included introducing SUs to the TEQ at ward admission, explaining ‘this is what you should expect’ as an inpatient, with opportunity to give voice to their experiences by completing the TEQ at discharge. Regular discussion of the TEQ in community meetings helped SUs and nurses become aware of its improvement role and the need for completion at discharge. Another successful idea was use of ‘you said, we did’ notices to publicize actions taken on a ward in response to TEQ data. There was further benefit if nurse directors/senior clinicians recognized the value of these agents of change, advocating their ideas and recommending and distilling their use across other wards.

However, there were reported to be difficulties of sustaining implementation efforts if an agent of change left, with no immediate successor. This was seen as particularly problematic if the intervention had not become successfully embedded into regular practice and ward culture.

[Ward manager] was the torch carrier but when they left it hadn't become embedded…If the champion leaves and there isn't a new champion soon enough it can quickly fall away. (Facilitative discussion meeting – Nurse Director/Senior Clinician, ID 01)

## DISCUSSION

Identifying the barriers and facilitators associated with implementing mental health nursing interventions is long seen as beneficial for development of effective implementation strategies (Forsner *et al*. 2010) and enhancing inpatient nursing care. This study used qualitative methods, in English acute mental health inpatient wards, to understand the barriers and enablers to implementation of the TEQ, an intervention to measure RMHN‐SU therapeutic engagement. To our knowledge, there is an absence of studies that have focused on the implementation of therapeutic engagement interventions in these settings and across multiple hospital organizations. This paper provides additional new knowledge in that the implementation process described took place during the COVID‐19 pandemic.

**FIG. 1:**
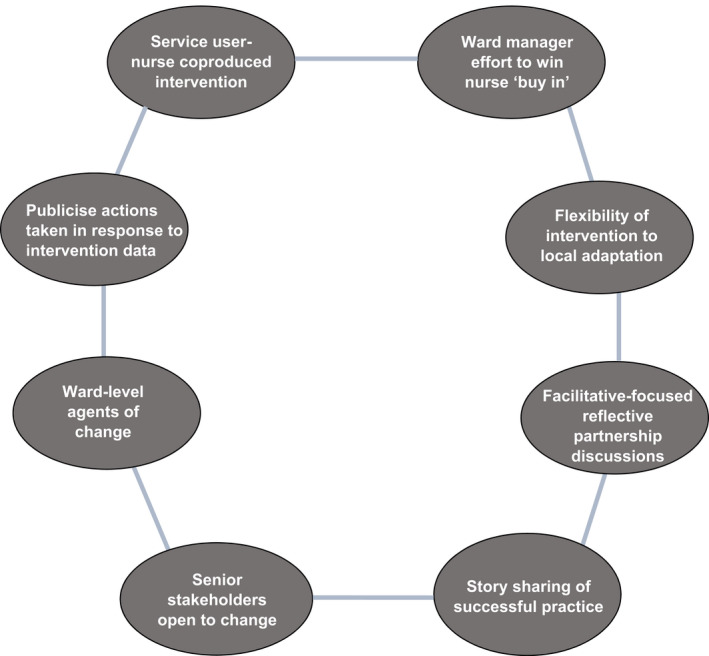
Conceptual model for facilitating implementation of the TEQ.

The merits of therapeutic engagement for inpatient recovery on acute mental health wards have long been recognized (Chambers 1992). Nonetheless, stimulating active nurse‐SU therapeutic engagement practice remains problematic (McAllister et al. 2021a). This study has shown that the TEQ is highly valued and welcomed by many stakeholders. However, a range of practical and perceptual factors impeded implementation. Addressing these issues should help support implementation. Our study suggests several enablers for successful implementation, brought together in a conceptual model (Fig. 1) that builds on the PARIHS framework, used as an organizing framework for data analysis and interpretation.

The PARIHS framework posits that an intervention that is evidence based provides an advantage for implementation (Kitson *et al*. 1998), and this was supported by our findings. Nurse directors/senior clinicians frequently mentioned their trust in the TEQ because of its empirical background and for being co‐produced with SUs and nurses. This credible evidence‐base provided them with the justification to recommend and support its implementation. Furthermore, there were stakeholders who mentioned being confident in using TEQ data at the macro level (in reporting to national quality monitoring organizations), meso level (to complement organizational strategic objectives), and micro level (to support ward‐based quality improvement and/or training initiatives).

Nonetheless, our study identified that contextual factors played a major role in impeding staff engagement with the intervention and its uptake at the ward level. The TEQ was implemented during a time of considerable upheaval for the sites involved, with severe ongoing clinical pressures associated with COVID‐19. Study data highlighted how many of the existing contextual challenges for intervention implementation in the acute inpatient setting reported in the literature (Raphael *et al*. 2021) were greatly magnified by the pandemic, most notably, time pressures, task‐oriented cultures, and staff shortages. Stakeholders reported difficulties in combining infection control measures with maintaining a therapeutic ward environment, which is in line with international evidence on the impacts of COVID‐19 in mental health inpatient settings (Rains *et al*. 2021). The study also revealed some staff resistance to TEQ data collection, particularly among staff who perceived this as performance monitoring.

The ability of suitable facilitation to ameliorate poor contexts for intervention implementation has been reported in the literature (Sandstrom *et al*. 2015) and is evident in our study data. Nurse directors/senior clinicians played an important role in facilitating or containing implementation of the TEQ; the more effective change motivators encouraging ward staff to discuss barriers and how they might be overcome. This is in line with existing evidence highlighting how staff in acute mental health leadership roles influence ward staff motivation to engage in change delivery (Laker *et al*. 2014). Yet in this study change leadership was not confined to nurse directors/senior clinicians. In some sites, ward‐level agents identified and championed changes that helped facilitate implementation, especially promotional initiatives among staff, and SUs and their families.

Facilitative discussion meetings among stakeholders and researchers also played a valuable role in addressing context‐related implementation barriers. The important contribution of conversations in facilitating intervention success has been identified in the literature (Jordan et al. 2009). In our study, the conversations were frequently distinguished by their informal and non‐hierarchical character, combining a reflective, ‘think‐aloud’ approach. This seemed to allow participants to describe their thoughts and opinions about the intervention implementation and to raise ideas for addressing obstacles. Used in facilitation, reflection has been shown to lead to critical questioning of processes and structures that can prompt practice change (Berta *et al*. 2015).

A further enabler was flexibility of the intervention. Flexibility was feasible because of clarity in defining the intervention's ‘hard core’ components (Greenhalgh *et al*. 2004). While these components were consistently implemented across sites, it was possible for the secondary components to be adapted and shaped to organizational contextual factors. One example was flexibility regarding time of completion of the TEQ. Another example was use of the TEQ as a training resource, supplementary to its designated use of measuring therapeutic engagement, thereby helping overcome staff resistance due to poor therapeutic engagement know‐how. This is congruent with evidence on the importance of intervention flexibility in helping overcome capability barriers (Raphael et al. 2021).

### Limitations

Study sites were self‐selecting which could have introduced some bias as local contextual factors associated with these sites may have differed from those of sites that did not participate. However, longitudinal data collection from multiple organizations across different English regions is likely to have minimized this bias. One of the study researchers [MC] led development of the TEQ which may have influenced their data interpretation. We tried to mitigate this by the team approach to analysis and drafting of the manuscript. Additionally, this study is limited by its focus on the nurse perspective; a further study is planned to evaluate the SU perspective.

## CONCLUSION

Policymakers and nursing leaders in many countries are considering strategies to integrate therapeutic engagement into acute mental health inpatient care to improve safety and quality. This paper offers insights on the factors to support successful implementation of the TEQ, a tool with the potential to measure and encourage therapeutic engagement. Our study has shown that the TEQ as an evidence‐based intervention co‐produced with SUs and nurses was welcomed and respected by stakeholders. However, a range of perceptual and practical barriers inhibited the implementation process. Addressing these issues can help support implementation of the TEQ in acute mental health inpatient wards, with some transferability to implementation of other interventions in these settings.

### RELEVANCE FOR CLINICAL PRACTICE

Successful implementation of the TEQ into clinical practice is feasible. However, the challenges faced in translating this intervention into a non‐receptive context suggest several practice implications for others implanting the TEQ or other interventions into the acute mental health inpatient environment. First, effort put into winning nurse ‘buy‐in’, for example by identifying and supporting ward‐level agents of change. Second, encouraging reflective and facilitative conversations among stakeholders. Third, utilizing the flexibility inherent in the intervention to create fit with local clinical need.

## FUNDING STATEMENT

Francesca Taylor and Mary Chambers are supported by the National Institute for Health Research (NIHR) Applied Research Collaboration (ARC) South London (NIHR ARC South London) at King's College Hospital NHS Foundation Trust. The views expressed are those of the authors and not necessarily those of the NIHR or the Department of Health and Social Care.

## Data Availability

The data that support the findings of this study are available from the corresponding author upon reasonable request.
